# Tapentadol is effective in the management of moderate-to-severe cancer-related pain in opioid-naïve and opioid-tolerant patients: a retrospective study

**DOI:** 10.1007/s00540-020-02821-8

**Published:** 2020-07-09

**Authors:** Shoichiro Sazuka, Toshiya Koitabashi

**Affiliations:** Department of Palliative Care Medicine, Ichikawa General Hospital, Tokyo Dental College, 5-11-13 Sugano, Chiba, Ichikawa-city 272-8513 Japan

**Keywords:** Tapentadol, Cancer pain, Neuropathic pain, Japan

## Abstract

**Purpose:**

Tapentadol is a dual-acting mu-opioid receptor agonist and noradrenaline reuptake inhibitor with non-inferior analgesic efficacy to oxycodone and better gastrointestinal tolerability than full mu-opioid receptor agonists. Tapentadol is approved for cancer pain in Japan; however, real-world evidence on tapentadol’s effectiveness and safety for cancer-related pain in Japan is limited.

**Methods:**

This retrospective study evaluated the effectiveness, safety, and tolerability of tapentadol (by patient type—opioid-naïve and opioid-tolerant) in 84 patients with moderate-to-severe cancer pain at Ichikawa General Hospital between September 2014 and August 2016.

**Results:**

Almost 93% of patients achieved clinically relevant pain relief within 4 days (median). Over 90% of patients with neuropathic pain or mixed pain and all patients with nociceptive pain were responders. Pain intensity significantly decreased from baseline through to the end of maintenance period in opioid-naïve and opioid-tolerant patients. No patients discontinued tapentadol due to serious adverse events. No opioid-naïve patients experienced nausea or vomiting during tapentadol treatment. Only three opioid-tolerant patients experienced nausea which was considered to be related to tapentadol.

**Conclusion:**

Tapentadol is effective and well tolerated in opioid-naïve and opioid-tolerant patients with cancer pain of varying pathophysiology, including those with nociceptive and/or neuropathic components. Tapentadol may be considered for first-line use in moderate-to-severe cancer-related pain.

## Introduction

Pain affects up to 70% of patients with cancer, especially those in the advanced stages of the disease [1]. Even after completing anti-cancer treatment, more than 30% of patients suffer from pain that requires treatment [2]. Cancer pain often involves a “mixed” pain type arising from both nociceptive and neuropathic pain [3]. The prevalence of cancer-related neuropathic pain is variable and ranges between 20 and 40% [4–7], whereas that for cancer-related mixed pain is reported to be up to 40% [4].

Neuropathic pain is defined by the International Association for the Study of Pain as “pain that arises directly from a lesion or diseases affecting the somatosensory system” [8]. Neuropathic pain is diagnosed using sensory examinations and diagnostic tests such as neuroimaging or neurophysiological tests to confirm a lesion or disease that may contribute to neuropathic pain [9]. Pharmacological options for cancer-related neuropathic pain include treatment with opioids, non-opioids, and adjuvant therapies such as tricyclic antidepressants or anticonvulsants [10]. Nevertheless, neuropathic pain remains a challenge to treat as effective management hinges on the reliable diagnosis, detection, and selection of the appropriate pharmacological agent by the primary physician [10, 11].

Opioids are the cornerstone in the treatment of moderate-to-severe cancer-related pain [12]. Opioids, although effective at alleviating cancer pain, may cause adverse effects such as constipation, sedation, and pruritus [13]. Previous exposure to opioids may also influence analgesic response and adverse effects. Equianalgesic doses of opioids have shown to induce more adverse effects with less analgesia in opioid-tolerant patients compared to opioid-naïve patients [14]. Guidelines by the European Society for Medical Oncology [12] and the Japanese Society of Palliative Medicine [15] recommend opioid switching or rotation to improve analgesic response or minimize the severity of adverse effects.

Tapentadol is a centrally acting mu-opioid receptor agonist and noradrenaline reuptake inhibitor [16]; both mechanisms of action contribute to its anti-nociceptive and anti-neuropathic pain effects [17–19]. Tapentadol has demonstrated to be non-inferior to oxycodone [20] and morphine [21] at reducing pain intensity with better gastrointestinal tolerability in patients with cancer pain. Improved gastrointestinal tolerability with tapentadol may be attributed to its dual mechanism of action and weaker affinity for the mu-opioid receptor compared to other full mu-opioid receptor agonists [19, 22]. Furthermore, tapentadol has been studied in both opioid-naïve and opioid-tolerant patients with pain as a direct consequence of cancer or from anti-cancer treatment [16]. Opioid switching to tapentadol has shown to be practical for improving pain relief and reducing the severity of adverse effects [23, 24]. Despite its approval for cancer pain in Japan in 2014, there is paucity of real-world evidence on the efficacy, safety, and tolerability of tapentadol in cancer pain management. Thus far, only one real-world study has studied the effectiveness and safety of tapentadol in Japanese patients with cancer-related pain that is unresponsive to first-line opioid therapy [24].

The present study aimed to evaluate the effectiveness, safety, and tolerability of tapentadol by patient type (opioid-naïve and opioid-tolerant) in Japanese patients with moderate-to-severe cancer pain.

## Methods

### Study population

We retrospectively evaluated 92 patients with cancer pain treated with tapentadol at Ichikawa General Hospital in Japan between September 2014 and August 2016. The study included opioid-naïve or opioid-tolerant patients who fulfilled the following criteria: diagnosed with moderate-to-severe cancer pain (numerical rating scale; NRS ≥ 4); required opioids for cancer pain control; did not require adjuvant analgesics; and dissatisfied with the cancer pain relief from current analgesic treatment.

Opioid-naïve patients were those not receiving opioid analgesic on a daily basis and, therefore, have not developed tolerance to opioids. Opioid-tolerant patients were those already treated with other opioid analgesics—i.e., at least 25 mcg/h fentanyl patch, at least 30 mg of morphine daily, at least 20 mg of oral oxycodone daily, or an equianalgesic dose of another opioid for a week or longer. Neuropathic pain was diagnosed based on a history of relevant neurological lesion, clinical evaluation of pain distribution that was anatomically consistent with the suspected location of the lesion and the existence of allodynia or hypoesthesia, and objective diagnostic tests (such as computed tomography and/or magnetic resonance imaging) confirming the presence of nerve lesion [25]. The study was approved by the research ethics committee of Ichikawa General Hospital (reference I 14-45R; approved on 24th Mar 2017). Patients were given the opportunity to opt out.

### Treatment

Opioid-naïve patients were prescribed an initial tapentadol dose of 25 or 50 mg twice daily (50–100 mg/day). Opioid-tolerant patients were prescribed an initial tapentadol dose of 50–400 mg/day. Tapentadol doses were calculated based on previous opioid consumption, and at a conversion ratio of 5:1 (tapentadol:oxycodone). Patients discontinued previous opioid analgesic treatment prior to receiving their first dose of tapentadol.

Oral immediate release oxycodone (5 or 10 mg) was available as rescue medication for breakthrough pain without limit on the number and timing of doses per day. Following stable dosing of tapentadol for at least 3 days, tapentadol doses were titrated at physician’s discretion. Tapentadol doses could be increased in increments of either 50 or 100 mg, up to a maximum of 400 mg/day for opioid-naïve patients or 600 mg/day for opioid-tolerant patients.

### Study endpoints

#### Effectiveness

Pain intensity was assessed by physicians or nurses at the following time-points: baseline, initial pain relief, and maintenance period. Patients rated their pain intensity using the NRS from 0 (no pain) to 10 (worst pain imaginable). Patients entered the maintenance period after achieving satisfactory pain relief and once tapentadol titration was completed. Satisfactory pain relief was defined as not having taken any rescue medications for breakthrough pain more than twice daily during a 3-day treatment period with stable doses of tapentadol. Following tapentadol titration, clinically relevant pain relief was defined as having at least 50% reduction in NRS score from baseline; patients who had clinically relevant pain relief were considered “responders”.

#### Safety and tolerability

Safety assessments included recorded nausea and vomiting and serious adverse events. We specifically assessed nausea and vomiting, because these are common adverse effects of opioid treatment. Data on constipation were not collected in this study, because prophylactic laxatives had been already prescribed or were prescribed at the time of tapentadol initiation in most patients. Reasons for discontinuation of tapentadol were also assessed.

### Statistical analyses

Demographics, patient characteristics, and effectiveness and safety endpoints were summarized using descriptive statistics. The effectiveness and safety endpoints were also evaluated by patient type (i.e., opioid-naïve and opioid-tolerant). Pain intensity scores between time-points (baseline vs. initial pain relief; initial pain relief vs. maintenance period) were compared using the two-way analysis of variance test. Discrete variables were compared using the Friedman test and post hoc Bonferroni correction. Statistical analyses were performed using Microsoft Office Excel 2013 (Los Angeles, CA, USA). A *P* value of less than 0.05 was considered to be statistically significant.

## Results

### Patient demographics and characteristics

A total of 92 patients entered the study. Eight patients had oral intake difficulty due to general physical health deterioration and were excluded from the analysis, leaving 84 patients for analysis. Patient demographics and characteristics are summarized in Table 1. More than half (59.5%) of the study population were male and the mean (standard deviation, SD) age of patients was 66.2 (11.5) years. The majority of patients were opioid-tolerant (*n* = 55; 65.5%); patients were previously treated with oxycodone (*n* = 27; 32.1%), tramadol (*n* = 20; 23.8%), fentanyl (*n* = 6; 7.1%), or morphine (*n* = 2; 2.4%). Among the opioid-tolerant patients, only four patients received more than 120 mg/day of morphine equivalent dose of opioid prior to switching to tapentadol: two patients received 100 mg/day of oxycodone and two patients received 120 mg/day of oxycodone. Approximately 41, 37, and 23% of patients were assessed as having mixed pain, neuropathic pain, and nociceptive pain, respectively.

**Table 1:** Patient demographics and characteristics (N = 84)

	All patients (*N* = 84)
Age (years)	66.2 (11.5)
Gender, *n* (%)	
Male	50 (59.5)
Female	34 (40.5)
Body mass index	20.9 (3.2)
Height (cm)	159.1 (8.2)
Body weight (kg)	53.3 (10.3)
Tumor origin, *n* (%)	
Lung	25 (29.8)
Renal	21 (25.0)
Hepatobiliary	10 (11.9)
Gynecological	10 (11.9)
Hematological	6 (7.1)
Others	12 (14.3)
Eastern Cooperative Oncology Group Performance Status, *n* (%)	
1	21 (25.0)
2	27 (32.1)
3	26 (31.0)
4	10 (11.9)
Previous opioid analgesic treatment, *n* (%)	
Oxycodone	27 (32.1)
Tramadol	20 (23.8)
Fentanyl	6 (7.1)
Morphine	2 (2.4)
None^a^	29 (34.5)
Pain type, *n* (%)	
Mixed pain^b^	34 (40.5)
Neuropathic pain	31 (36.9)
Nociceptive pain	19 (22.6)

Data are presented as mean (SD), unless otherwise stated

^a^Opioid-naïve

^b^Neuropathic and nociceptive pain

### Effectiveness

**Fig. 1:**
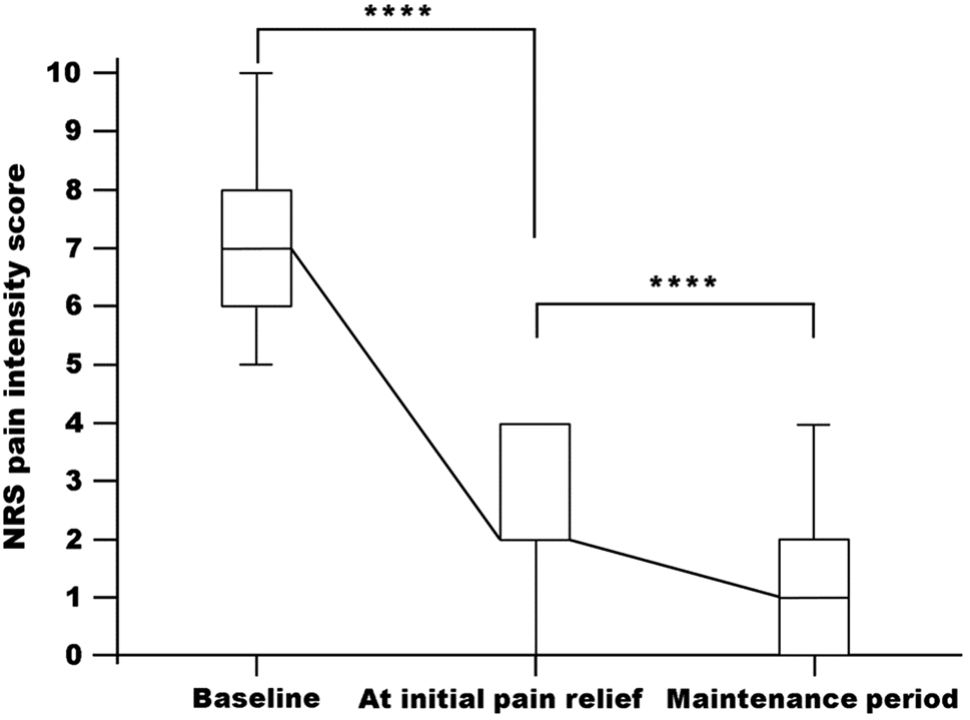
Median numerical rating score (NRS) pain intensity scores over time in the overall study population. Boxes indicate 25th (bottom) and 75th (top) percentiles; horizontal line within boxes indicates median; error bars indicate 10th–90th percentiles. ****P < 0.0001

Following tapentadol administration, 92.9% (78 of 84) of all patients achieved clinically relevant pain relief (i.e., at least 50% reduction in NRS score from baseline) within a median (range) of 4 (1–13) days. The proportion of responders in patients with mixed, nociceptive, and neuropathic pain were 91.1% (31/34), 100% (19/19), and 90.3% (28/31), respectively. The median (range) NRS score decreased significantly from 7 (4–10) at baseline to 2 (0–6) at initial pain relief (*P* < 0.0001; Fig. 1). At the time of initial pain relief, the median (range) daily dose of tapentadol was 100 (50–300) mg. During the maintenance period, the increase in median (range) daily dose of tapentadol to 200 (100–600) mg resulted in further pain relief in 56 of 78 (71.8%) of patients. In these 56 patients, the median (range) NRS score decreased from 2 (0–6) at initial pain relief to 1 (0–5) at the end of the maintenance period (*P* < 0.0001; Fig. 1).

In six of 84 patients, tapentadol was started in median (range) daily dose of 75 (50–200) mg and increased to daily dose of up to 100–400 mg. In these six patients, the median (range) NRS score was 6 and remained unchanged from baseline to the end of the maintenance period; they were considered non-responders and were subsequently switched to other opioid analgesics such as oxycodone or transdermal fentanyl.

### Effectiveness in opioid-tolerant patients

Of 55 opioid-tolerant patients, 52 patients (94.5%) achieved clinically relevant pain relief within a median of 3 days and were responders. The median (range) NRS score decreased from 7 (4–10) at baseline to 3 (0–6) at initial pain relief. The median (range) NRS scores decreased from 3 (0–6) at initial pain relief to 1 (0–5) at the end of the maintenance period.

### Effectiveness in opioid-naïve patients

**Fig. 2:**
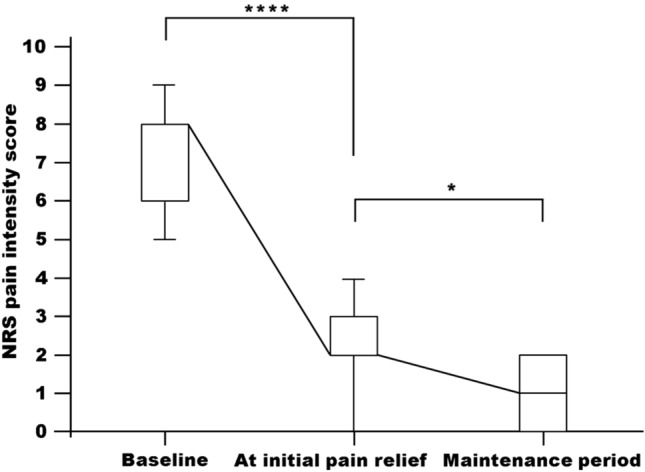
Median numerical rating score (NRS) pain intensity scores over time in opioid-naïve patients. Boxes indicate 25th (bottom) and 75th (top) percentiles; horizontal line within boxes indicates median; error bars indicate 10th–90th percentiles. ****P < 0.0001; *P < 0.05

Of 29 opioid-naïve patients, 26 patients (89.7%) achieved clinically relevant pain relief within a median (range) of 3 (1–11) days and were responders. The median (range) NRS scores decreased from 8 (4–10) at baseline to 2 (0–4) at initial pain relief (*P* < 0.0001; Fig. 2). At the time of initial pain relief, the median (range) daily dose of tapentadol was 100 (50–200) mg. During the maintenance period, the increase in median (range) daily dose of tapentadol to 200 (75–400) mg resulted in further pain relief in 19 of 26 (73.1%) patients. In these 19 patients, the median (range) NRS scores decreased from 2 (0–4) at initial pain relief to 1 (0–2) at the end of the maintenance period (*P* < 0.05).

### Safety and tolerability

At baseline, nausea was observed in 22 of 84 (26.2%) patients, of whom 19 were opioid-tolerant and three were opioid-naïve. Nausea disappeared in nine of 19 (47.3%) patients who had exhibited nausea caused by previous strong opioids. No opioid-naïve patients experienced nausea or vomiting during tapentadol treatment. Only three opioid-tolerant patients experienced nausea which was assessed to be related to tapentadol by the physician. During the maintenance period, three patients discontinued tapentadol due to general health deterioration caused by disease progression. No patients discontinued tapentadol or had dose reduction due to serious adverse events, and none exhibited withdrawal symptoms. There were no somnolence or delirium which required treatment discontinuation or dose reduction.

## Discussion

This study aimed to evaluate the effectiveness, safety, and tolerability of tapentadol in Japanese patients with moderate-to-severe cancer pain. The findings revealed that tapentadol was effective at relieving cancer pain and well tolerated in both opioid-tolerant and opioid-naïve patients during the study period. More than 90% of patients with neuropathic pain or mixed pain, and all patients with nociceptive pain were responders.

Previous exposure to opioids, among others such as pain type (i.e., neuropathic pain), and progressive disease, can influence a patient’s response to analgesic treatment [14, 26] and, hence, efficacy outcomes. In the present study, we observed a significant reduction in pain intensity scores from baseline through to the maintenance period in both opioid-naïve and opioid-tolerant patients. These findings suggest that tapentadol was effective at alleviating moderate-to-severe cancer pain regardless of previous exposure to opioids and corroborate those from Kress and colleagues [21], who reported that tapentadol was non-inferior to morphine in managing moderate-to-severe cancer pain in both opioid-naïve and opioid-tolerant patients. Although our study did not have an active comparator to compare the effectiveness for pain relief, it is notable that pain was significantly relieved in patients who switched to equivalent dose of tapentadol.

Both ascending and descending pathways are implicated in pain and are common sites of actions of analgesics [17, 27]. Tapentadol has a dual mechanism of action as a mu-opioid receptor agonist of the ascending pathway and as a noradrenaline reuptake inhibitor of the descending pathway [17, 18]. Noradrenaline plays an important role in pain modulation, particularly that of neuropathic pain [18, 28]. In rat models of spinal nerve ligation, tapentadol administration induced a stronger noradrenergic inhibition over mu-receptor agonism [29]. Furthermore, both α2-adrenoceptor agonism and mu-receptor agonism were almost completely reversed by selective antagonists, atipamezole and naloxone, respectively, highlighting the exclusive synergy between both noradrenaline reuptake inhibition and mu-opioid receptor agonism [29].

In the present study, 41, 37, and 23% of patients were assessed as having mixed pain, neuropathic pain, and nociceptive pain, respectively. All patients with nociceptive pain and more than 90% of patients with neuropathic pain or mixed pain were responders. These findings highlight tapentadol’s effectiveness not only for cancer-related nociceptive pain, but also neuropathic pain and the results are mirrored in other studies [21, 24, 30–32].

Tapentadol’s additional mechanism of action (i.e., inhibition of noradrenaline reuptake via α2-adrenoceptors) [28] means that it is able to alleviate both nociceptive pain and neuropathic pain [17–19]. This unique characteristic distinguishes tapentadol from most other strong/WHO step III opioids (i.e., morphine and fentanyl) that are mainly mu-opioid receptor agonists [33]. Indeed, tapentadol has demonstrated effectiveness and tolerability in Japanese patients with cancer-related neuropathic pain that was unresponsive to previous opioid therapy [24]. Other studies supporting tapentadol for cancer-related neuropathic pain have been mainly conducted in European patients [21, 30]. Tapentadol has also shown to be a suitable option for chemotherapy-induced neuropathic pain [31]. By targeting both nociceptive and neuropathic pain, tapentadol could improve treatment adherence and outcomes (e.g., quality of life), and minimize the potential for adverse effects [34].

Gastrointestinal side effects are commonly associated with opioid therapy [35]. In the present study, we evaluated the incidence of nausea and vomiting before and after tapentadol treatment and observed a favorable gastrointestinal tolerability, especially in opioid-naïve patients. Notably, no opioid-naïve patients experienced nausea or vomiting during tapentadol treatment and only three opioid-exposed tolerant patients experienced nausea which was considered to be related to the treatment. No patients discontinued tapentadol during treatment. We acknowledge that we are unable to draw conclusive statements regarding tapentadol’s safety as opioid-induced adverse effects extend beyond nausea and vomiting. Nevertheless, published literature have demonstrated the acceptable safety and tolerability profile of tapentadol in patients with cancer-related pain [21, 24, 30–32]. Besides, tapentadol has also demonstrated better gastrointestinal tolerability due to its dual mechanism of action and weaker affinity for the mu-opioid receptor compared to other full mu-opioid receptor agonists [19, 22].

This study also presents some inherent limitations. For example, the retrospective study design limited our scope of analysis and interpretations as pertinent information (e.g., duration of previous opioid therapy, stable or progressive disease, etc.) were lacking due to the use of data originally recorded for other purposes. Additionally, having an active comparator would have provided a more balanced representation of tapentadol’s clinical effectiveness. Our study would also benefit from evaluating the reduction in pain intensity across subgroups of patients with different pain types. Furthermore, this was a single-center study where the size of the study population was small and all patients were treated at the same center; the results should, therefore, be interpreted with caution. However, it is notable that our results are consistent with those of other studies. Although these limitations are important, our study adds on to the evidence of the real-world use of tapentadol in Japanese patients with cancer pain, and provides valuable insights into the use of tapentadol in a different ethnic patient population compared with those from previously conducted studies.

In conclusion, our study showed that tapentadol is effective and well tolerated in opioid-naïve and opioid-tolerant Japanese patients with cancer pain of varying pathophysiology, including those with a nociceptive and/or neuropathic components. Tapentadol is a valuable option for opioid-naïve or opioid-tolerant patients with inadequate pain relief or intolerable adverse effects with other opioids.

## Data Availability

The data that support the findings of this study are available from the corresponding author, TK, upon reasonable request.
